# Evaluation of agreement of IOP measurements by Tono-Vera tonometer to Goldmann applanation tonometry

**DOI:** 10.3389/fopht.2024.1441343

**Published:** 2024-08-16

**Authors:** Charles R. Niles, Alexander R. Crinzi, Russell Bonaventura, David A. Taylor

**Affiliations:** ^1^ Ophthalmology Associates of WNY, Williamsville, NY, United States; ^2^ Department of Ophthalmology, State University of New York at Buffalo, Buffalo, NY, United States; ^3^ Reichert, Inc., Buffalo, NY, United States

**Keywords:** tonometer, tonometry, IOP, Goldmann, Tono-Vera, GAT, Rebound

## Abstract

**Purpose:**

To evaluate the accuracy of the new Tono-Vera rebound tonometer (Reichert Inc, Buffalo, NY) compared to Goldmann Applanation Tonometry.

**Methods:**

This prospective, observational, cross-sectional study was designed in accordance with ANSI Z80.10-2014 and ISO 8612-2009 guidelines for tonometer comparison. Intraocular Pressure (IOP) was measured by Goldmann Applanation and Tono-Vera on 160 eyes of 160 subjects. Corneal Astigmatism and Central Corneal Thickness were also measured. A single investigator (CN) conducted all measurements. The average of two measurements from each tonometer was used in the analysis. Bland-Altman plots, total least squares regression analysis, and simple linear regression were used to evaluate agreement between the tonometers.

**Results:**

Average IOP values from Goldmann Applanation and Tono-Vera were not significantly different (19.17 and 19.03 respectively, p=0.40, paired t-test). The total least squares regression analysis indicated strong agreement between the two tonometers (slope +0.97, offset +0.49 mmHg, standard deviation 2.11 mmHg). There were 2 IOP measurement pairs that exceeded the ± 5 mmHg limits of agreement required in ANSI Z80.10-2014 and ISO 8612-2009, which is within the range of acceptability specified in the standards.

**Conclusion:**

We evaluated IOP measurements by Tono-Vera Rebound Tonometer vs Goldmann Applanation Tonometry for eyes with a wide range of IOP values and found no statistically significant differences in the results. Tono-Vera meets the requirements of ANSI Z80.10-2014 and ISO 8612-2009, demonstrating accuracy comparable to Goldmann tonometry.

## Introduction

Intraocular pressure (IOP) is the only modifiable risk factor for glaucoma, a worldwide leading cause of blindness (1). Despite numerous advances in diagnostic and treatment options over the past two decades, IOP reduction remains the only proven way to reduce rates of glaucoma progression (2). As such, regular and accurate IOP assessment continues to play a critical role in the diagnosis and management of glaucoma (3–5). Goldmann Applanation Tonometry (GAT) is considered the clinical “gold standard” for IOP measurement. In addition, international tonometer accuracy standards by ANSI (recognized by FDA) and ISO (recognized by many international regulators), use GAT as the reference standard for tonometer comparison (6, 7). Despite its widespread use and acceptance, GAT has several well documented limitations. Confounders such as Central Corneal Thickness (CCT), corneal hysteresis, and tear film affect the accuracy of GAT (8–10). Routine wear often causes calibration errors, which may go undetected by clinicians (11). In some patients IOP decreases due to the application of topical anesthesia (12). Repeat measurements may result in a “massage effect” that lowers the IOP (13). Operator influence and bias due to differing skill levels and techniques make GAT subject to high inter and intra operator variability (14). Especially when considering the increasing prevalence of obesity in modern western countries, GAT can be difficult to use and provide erroneous values as patients strain to get into position for measurement (15). Finally, particularly true in the post-COVID world, concerns related to contamination and disinfection are increasingly relevant (16).

In order to overcome at least some of the perceived limitations of GAT, numerous other tonometer technologies have been developed and have gained popularity over the years (17). Some tonometers are used in place of GAT, while others are considered “screening” devices and are commonly used in addition to GAT. Rebound tonometers have been on the market since the early 2000’s and have been adopted by many practitioners due to their ease of use, objective measurement results, and the fact that the method is well tolerated by patients (18). Tono-Vera (TV) (Reichert Inc, Buffalo, NY USA) is a new hand-held rebound tonometer. The principles of rebound tonometry have been described in detail elsewhere (19). Briefly, similarly to existing rebound tonometer technology on the market, Tono-Vera utilizes a lightweight, single-use, ferrous-shafted probe with a smooth, plastic contact tip (Ocu-Dot Tonometer Probe, Reichert, Inc, Buffalo, NY USA). The probe is propelled forward by a solenoid-induced magnetic field. The motion of the probe generates a voltage in the solenoid, which is recorded throughout the measurement process. IOP is derived from analyzing the velocity profile of the probe during the measurement. As the probe briefly contacts the cornea, its forward motion is stopped and ultimately reversed. The deceleration of the probe as it contacts the cornea and comes to a rest, represented by the measured voltage, is used determine the IOP. An internal calibration converts the voltage into an IOP value (displayed in mmHg on the instrument screen), which is intended to match GAT.

In order to reduce operator bias and the resultant measurement variability it causes, Tono-Vera features a unique camera-based, 3-dimensional positioning system referred to as “ActiView”. This system uses on-screen cues to guide the operator to the optimal position over the corneal apex at the correct distance. Once the alignment criteria are met the instrument measures automatically. Tono-Vera features two measurement modes. In “3+” measurement mode a minimum of 3 and a maximum of 6 successive measurements (based on the repeatability of the obtained values) are used to calculate the final IOP result. In “6-measurement” mode, 6 successive measurements are always made to calculate the final IOP result. 6-Measurement mode was used on all subjects for this study.

## Methods

This study was commissioned in preparation for an FDA 510k application for the Tono-Vera Tonometer and was prospectively registered with clinicaltrials.gov (NCT05345262). Approval for this study was obtained from Sterling Institutional Review Board. Eligible subjects were screened, enrolled, and evaluated according to the study protocol. Subjects were recruited from the study-site clinical population or referred to the Principle Investigator (PI) for the study by other local clinicians. Subjects meeting the inclusion criteria who provided written informed consent participated in the study. Measurements were conducted between April 2022 and September 2022. A single investigator (CN) conducted all of the Goldmann and Tono-Vera IOP measurements.

The study followed FDA Regulations relating to good clinical practice and clinical trials and protection of human volunteers (45 CFR Part 46, 21 CFR part 56), the ethical principles contained within the Declaration of Helsinki, and ISO 14155:2020 (Clinical investigation of medical devices for human subjects -Good clinical practice). The study was conducted to be in accordance with FDA Guidance for Tonometers (March 27, 2006), ANSI Z80.10-2014 (American National Standard for Ophthalmics –Tonometers) and ISO 8612-2009 (International Organization for Standardization, Ophthalmic instruments — Tonometers).

### Objectives

The primary objective of the clinical study was to demonstrate equivalence of the Tono-Vera tonometer (test device) to the GAT (reference device) in the measurement of IOP in accordance with ANSI Z80.10 2014 and ISO 8612-2009. In brief, these two standards require that the test tonometer match the reference tonometer within ± 5.0 mmHg in 95% of matched measurement pairs. Eyes are separated into low, medium, and high IOP categories (defined by the reference tonometer). Specifics regarding the requirements for pair-testing of the reference to test tonometer are provided in **Table 1**.

**Table 1:** ANSI Z80.10-2014 & ISO 8612-2009 requirements for paired testing of reference to test Tonometers.

IOP Range (mmHg)	Corneal Astigmatism (diopters)	Tolerance of Paired Differences (mmHg)	Minimum Number of Eyes
7 to 16	≤3D	±5	40*
>16 to < 23	≤3D	±5	40*
≥ 23	≤3D	±5	40*
7 to 16	>3D	±5	10**
>16 to < 23	>3D	±5	10**
≥ 23	>3D	±5	10**

ANSI, American National Standards Institute; ISO, International Organization for Standards; IOP, intraocular pressure.

*ANSI Z80.10 and ISO 8162 both require 40 eyes in each of the three IOP categories.

**ANSI Z80.10 requires 10 additional eyes per IOP category with greater than 3D astigmatism.

### Protocol

Participant inclusion criteria required that subjects must be between the ages of 18 and 90 years old, must able and willing to provide signed informed consent, and must be able to follow study instructions. Exclusion Criteria specified by the ANSI Z80 standard were: Subjects with only one functional eye, subjects with one eye having poor or eccentric fixation, subjects with central corneal thickness greater than 600 µm or less than 500 µm, subjects with corneal scarring or who have had corneal surgery (including laser refractive surgery), subjects with concomitant ocular diseases (including: microphthalmos, buphthalmos, nystagmus, keratoconus, severe dry eye syndrome, blepharospasm, any other corneal or conjunctival pathology or infection likely to confound the ability to obtain accurate IOP measurements), and contact lens wearers. In addition, subjects with known allergy to proparacaine or fluorescein were excluded. Glaucoma, suspicion of glaucoma, or presence of ocular hypertension were not exclusion criteria.

Trial participants were recruited from the study site (a private practice in Buffalo, NY, USA). Subjects were also able to be referred from other practices. The Principal Investigator (PI) or a designee identified potential subjects for the study from patient records and daily patient clinic visits. Subjects determined to be potentially eligible and who expressed interest in participating were consented and enrolled in the study. The protocol required that only one eye per patient be used in the analysis.

All subject measurements were made during a single visit to the study site. The following procedures were performed in the order listed:

1. Corneal Curvature by Auto-Refractor/Keratometer (Opto-Chek Auto Refractor Keratometer, Reichert Inc, Buffalo NY) (to determine amount of corneal astigmatism)
2. Slit lamp biomicroscopy (to ensure no exclusion criteria were present)
3. GAT (Reference Tonometer. Defines which IOP category the eye belongs to)
4. Tono-Vera (Test Tonometer)
5. Central Corneal Thickness by ultrasound pachymetry (iPac Pachymeter, Reichert Inc, Buffalo, NY) (to ensure selected subjects were between 500-600 microns per the requirements of ANSI Z80.10 and ISO 8621).

Both eyes were evaluated with the Auto-Refractor/Keratometer, slit lamp, and GAT. To compensate for the potential impact of corneal astigmatism, GAT was measured twice on each eye: once at 90-degrees and once at 180 degrees. The average of the two measurements was used as the GAT IOP value. Measuring with GAT at 90 and 180 and using the average as the reported IOP has been described and is a common method for nullifying the effects of corneal astigmatism (20–22). A new GAT tonometer (Haag-Streit, Switzerland) was used for the study and underwent calibration verification before the study and weekly during the study.

In order to ensure the widest possible range of IOP values, the eye to be included in the study was selected based on the following criteria:

- If only one eye qualifies as a high astigmatic (>3 Diopters of corneal astigmatism), select that eye.
- If the GAT pressures are unequal and ≥23 mmHg in the two eyes, select the eye with the higher IOP.
- If the GAT pressures are unequal and between >16 and <23 mmHg in the two eyes, select the eye with the higher IOP.
- If the GAT pressures are unequal and ≤16 mmHg in the two eyes, select the eye with the lower IOP.
- If the GAT pressures are in different IOP subranges in the two eyes, select the eye with the pressure in the subrange with fewer enrolled eyes.
- If the pressures in the two eyes are identical, randomly select an eye based on a coin toss or other valid binary randomization method.

The Tono-Vera (Test tonometer) was used in Automatic 6-measurement mode. In this mode, the on-screen alignment system of the tonometer guides the user to the optimal XYZ position over the apex of the cornea and automatically measures six times in rapid succession once the alignment criteria are met. Measurements were made on the selected eye no more than 3 minutes after the GAT measurement was completed. Two Tono-Vera measurements were obtained and averaged for the Tono-Vera IOP value. Pachymetry was measured last by iPac ultrasound pachymeter (Reichert, Inc. Buffalo, NY). Subjects with CCT under 500 or over 600 microns were excluded.

### Statistical methods

Data was analyzed per ANSI Z80.10-2014 section B9.3

a) A scatter plot of the test tonometer measurements (x-axis) versus the reference tonometer measurements (y-axis, scaled identically to x-axis) with the total least squares fitting line (orthogonal) and the y=x diagonal line drawn on the plot.

b) Results of the total least squares regression analysis of the data, including the number of paired results, estimated slope and intercept (offset of the regression), the standard deviation of the regression, the number of pairs in each IOP range and the number and percentage of pairs in each range that exceed the limit.

c) A method of differences plot (Bland-Altman) showing the paired differences versus the mean of the measured values of the pair for each of the IOP ranges given in **Table 1** with lines drawn on the plot indicating 5 mmHg greater than and 5 mmHg less than the zero difference axis.

In addition, a simple linear regression was used to calculate the correlation between GAT and Tono-Vera (r^2^).

For this publication, a paired t-test was used to compare Tono-Vera and GAT. A two-sample t-test was used to compare the differences between the Tono-Vera and GAT devices for eyes with low and high corneal astigmatism.

## Results

One Hundred sixty eyes (160) of 160 subjects were included in the study. Seventy-seven (77) of the eyes selected were right eyes and Eighty-three (83) were left eyes. There were 57 eyes in the low IOP (7-16 mmHg) category, 54 eyes in the medium IOP (>16 to <23 mmHg) category, and 49 eyes in the high IOP (≥23 mmHg) category based on the reference tonometer readings. 137 of the eyes measured were classified as low (<3 D) astigmatism. Patients were 55% Female. The study site was an ophthalmology practice with a large glaucoma population. As such, nearly all subjects (146) were documented as glaucoma, glaucoma suspect, or ocular hypertension (which were not exclusion criteria for the study). Patient characteristics are shown in **Table 2**.

**Table 2:** Patient characteristics.

N	160
Age (y)	69.7 ± 11.6 (22-88)
Sex (Female n %)	88 (55%)
IOP (GAT, mmHg)	19.17 ± 6.5 (7-43)
IOP (TV, mmHg)	19.03 ± 6.3 (8.5 – 39)
CCT (um)	553.4 ± 25 (500 – 600)
Corneal Astigmatism (D)	1.5 ± 1.6, (0 - 7.8)

IOP, intraocular pressure; GAT, Goldmann Applanation Tonometry; TV, Tono-Vera Tonometer; mmHg, millimeters of mercury; CCT, Central Corneal Thickness; um, microns.

### Accuracy of IOP (primary endpoint)

All but 2 of the 160 paired measurement differences were within the ±5 mm Hg tolerance specified in ANSI Z80.10 and ISO 8612. One outlier was observed in the medium IOP category and one in the high IOP category representing 1.85% and 2.04% of the matched pairs respectively, both in the <3D eye portion of the population. None of the >3D astigmatism eyes were classified as outlier measurements. ANSI Z80.10 and ISO 8612 permit up to 5% of matched pair measurements to reside outside of ± 5.0 mmHg (which equates to a maximum of 2 outliers per IOP category in this study). Tono-Vera satisfies this requirement. These results are summarized in **Table 3**.

**Table 3:** Results of Tono-Vera and GAT matched measurement pairs by IOP category.

IOP Range (mmHg) Defined By GAT		Astigmatism	N Eyes	Average GAT IOP (mmHg)	Average TV IOP (mmHg)	Measurement Pair Difference > ±5 mmHg	Percentage of Measurement Pair Differences > ± 5 mmHg
Low IOP	7 to 16	≤3D	49	12.7	13.3	0	0.00%
Medium IOP	>16 to < 23	≤3D	43	19.6	19.1	1	2.27%
High IOP	≥ 23	≤3D	45	27.4	26.7	1	2.17%
Low IOP	7 to 16	>3D	11	13.1	12.9	0	0.00%
Medium IOP	>16 to < 23	>3D	10	19.4	18.2	0	0.00%
High IOP	≥ 23	>3D	2	27.0	26.3	0	0.00%
Total			160	19.17	19.03	2	1.25%

IOP, intraocular pressure; GAT, Goldmann Applanation Tonometry; TV, Tono-Vera Tonometer.

**Figure 1:**
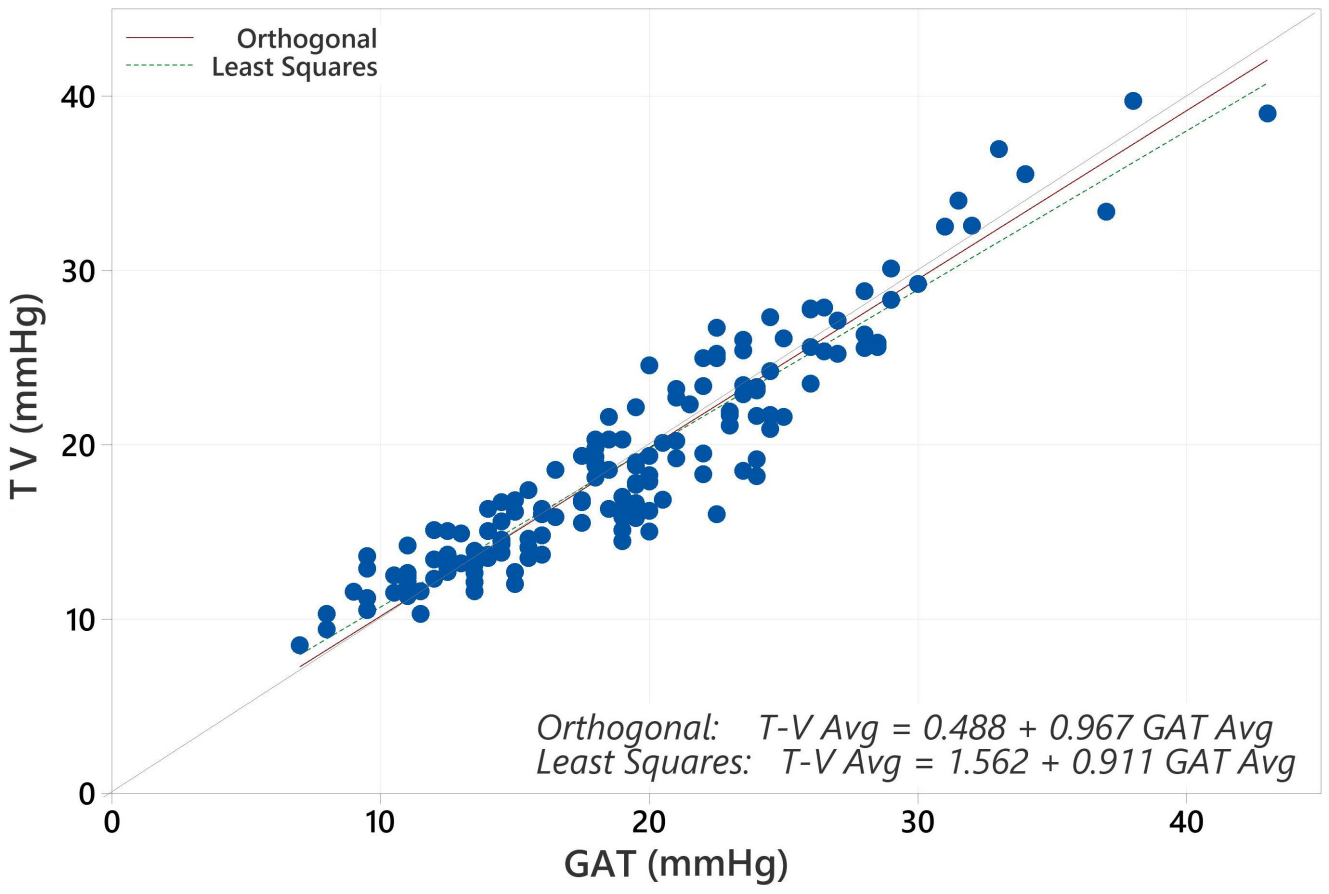
Total Least Squares Regression of Tono-Vera vs GAT for all 160 eyes.

**Figure 2:**
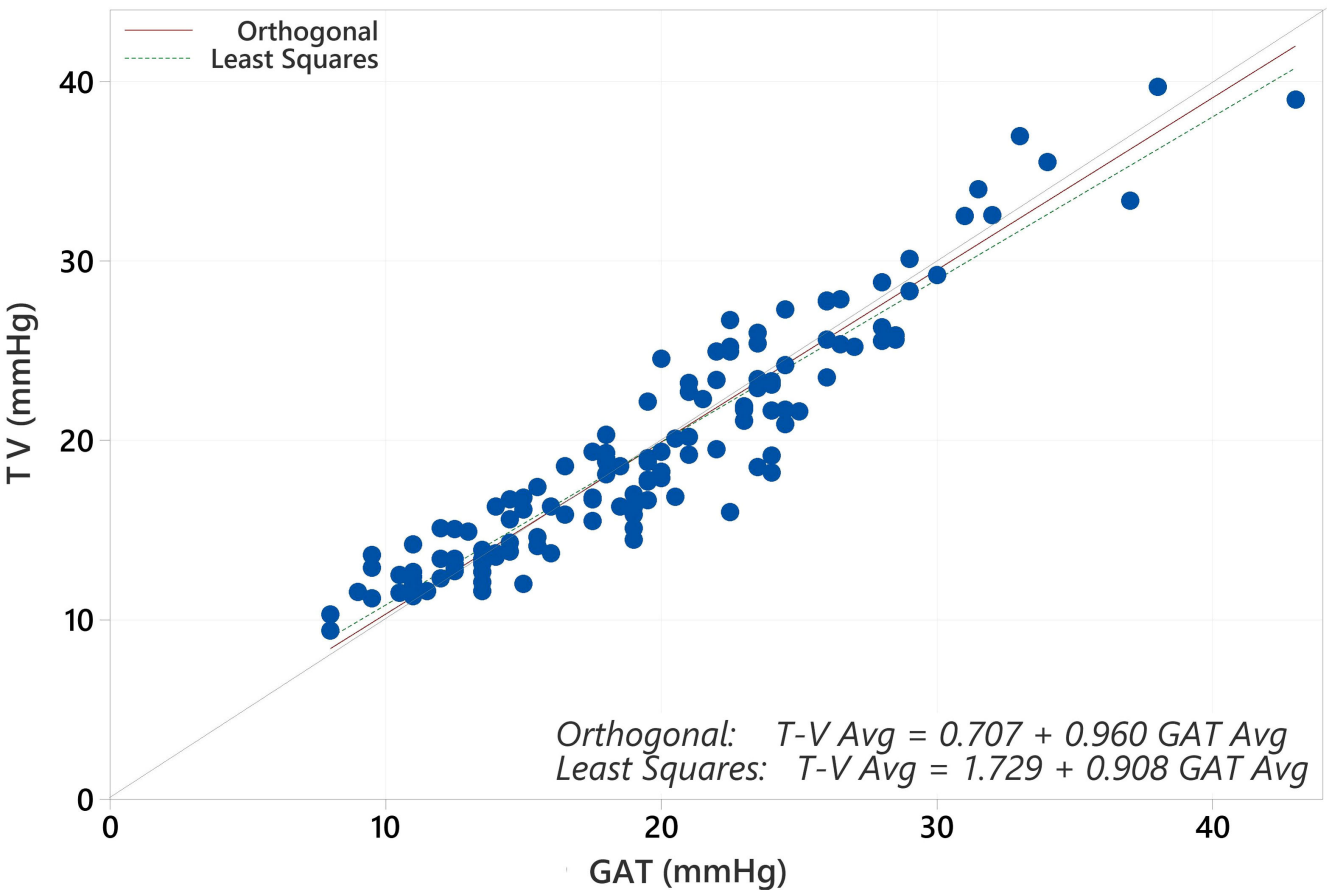
Total Least Squares Regression of Tono-Vera vs GAT for 137 low corneal astigmatism eyes.

**Figure 3:**
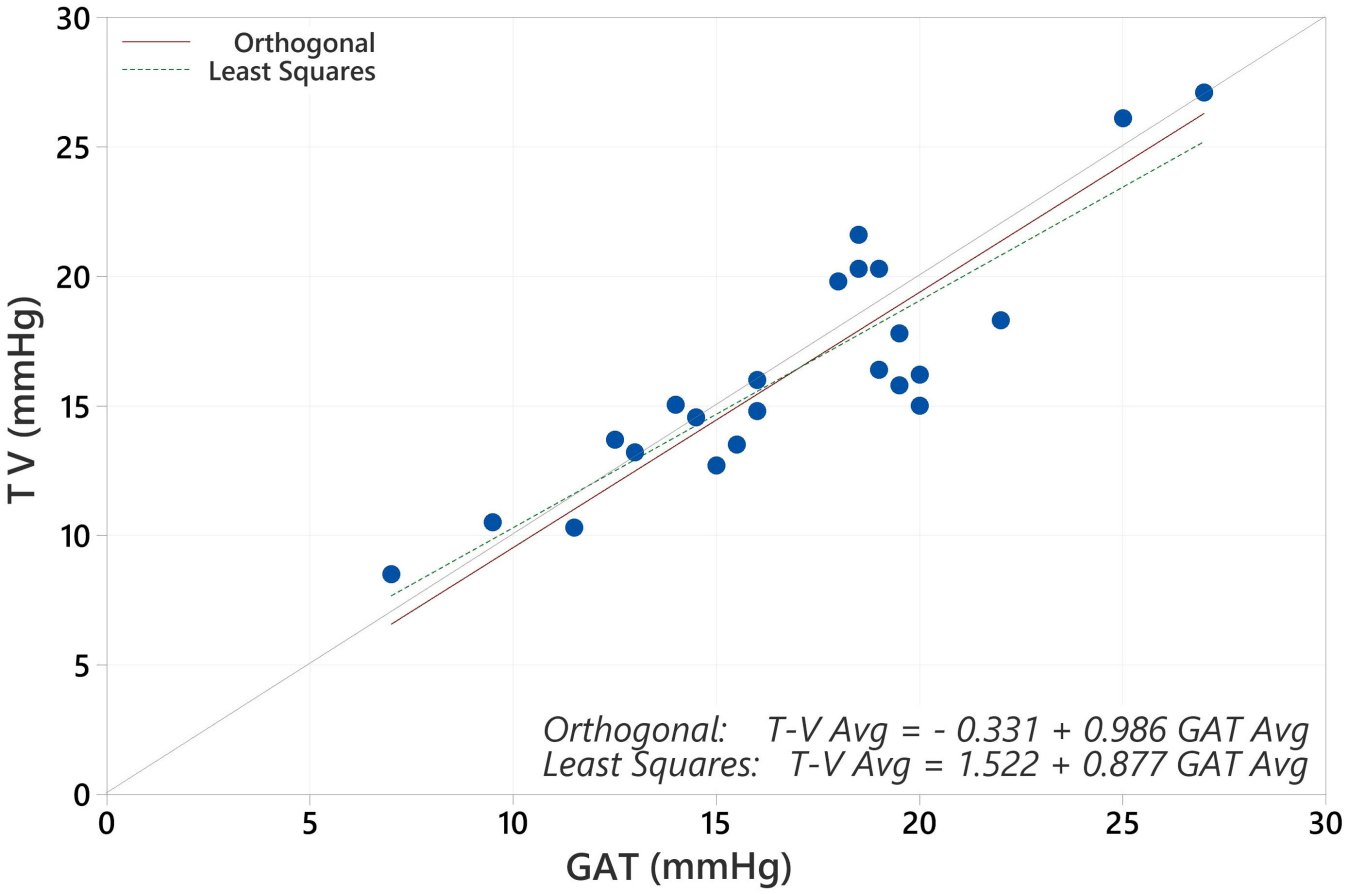
Total Least Squares Regression of Tono-Vera vs GAT for high corneal astigmatism eyes.

The average IOP for the population with GAT was 19.17 mmHg and for Tono-Vera was 19.03 mmHg (p=0.40, paired t-test). The correlation between GAT and Tono-Vera was r^2^ = 0.89. The Tono-Vera vs GAT Total Least Squares Regression (TLSR) plot for all data indicates excellent fit between Tono-Vera and GAT data throughout the range of measured IOP values with a slope of +0.97, offset of +0.49 mm Hg, and a standard deviation of 2.11 (**Figure 1**). **Figure 2** shows the TLSR for the 137 eyes with low astigmatism (≤3D). Here also we observed excellent fit between Tono-Vera and GAT IOP measurement data with a slope = +0.96, offset of +0.70 and a standard deviation of 2.17. **Figure 3** shows the TSLR for the 23 eyes in the high astigmatism (>3D) subgroup (slope = +0.99, and y-intercept of +0.33 and a standard deviation of 2.11).

**Figure 4:**
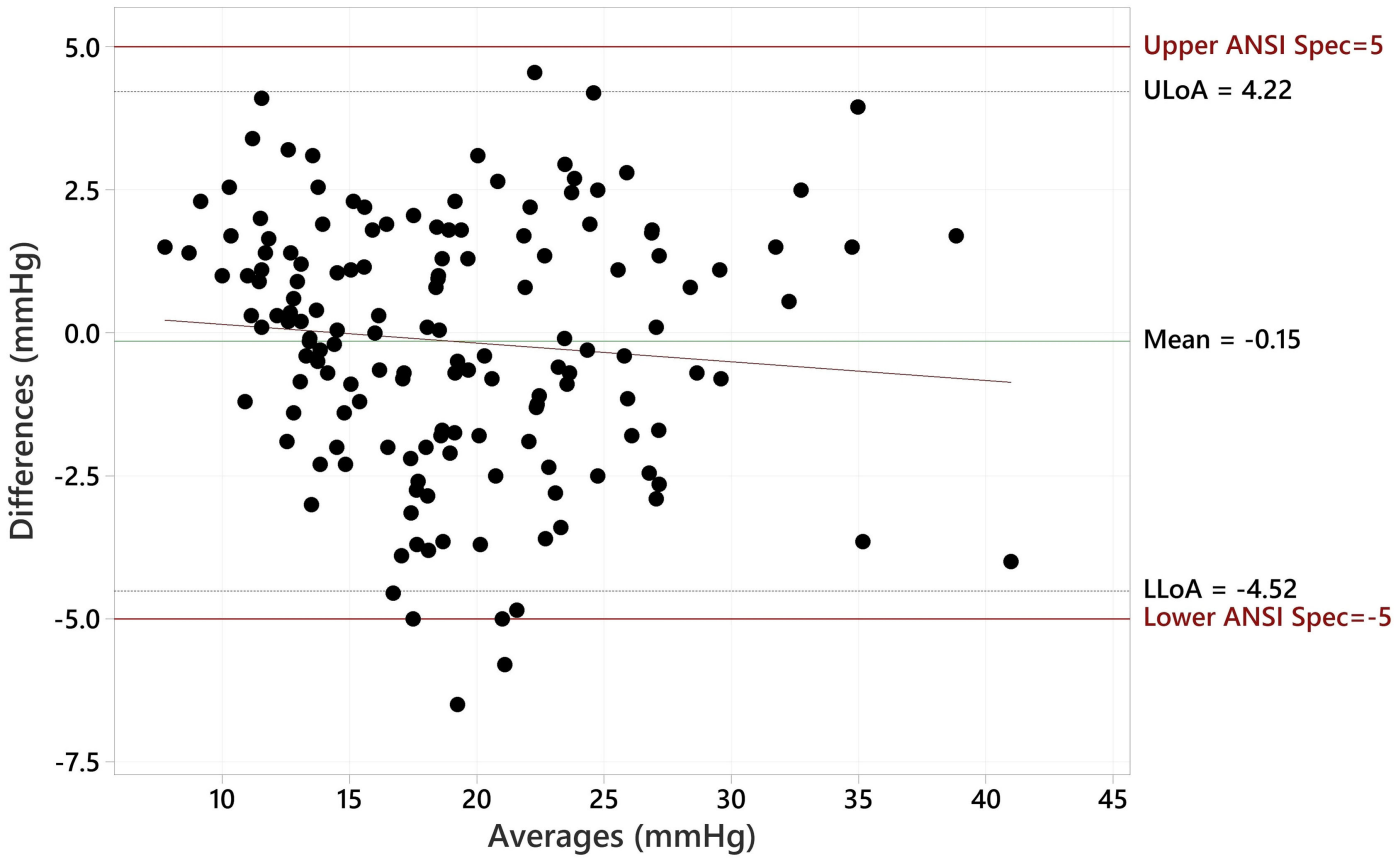
Bland-Altman Comparison of Tono-Vera to GAT for all 160 eyes.

**Figure 5:**
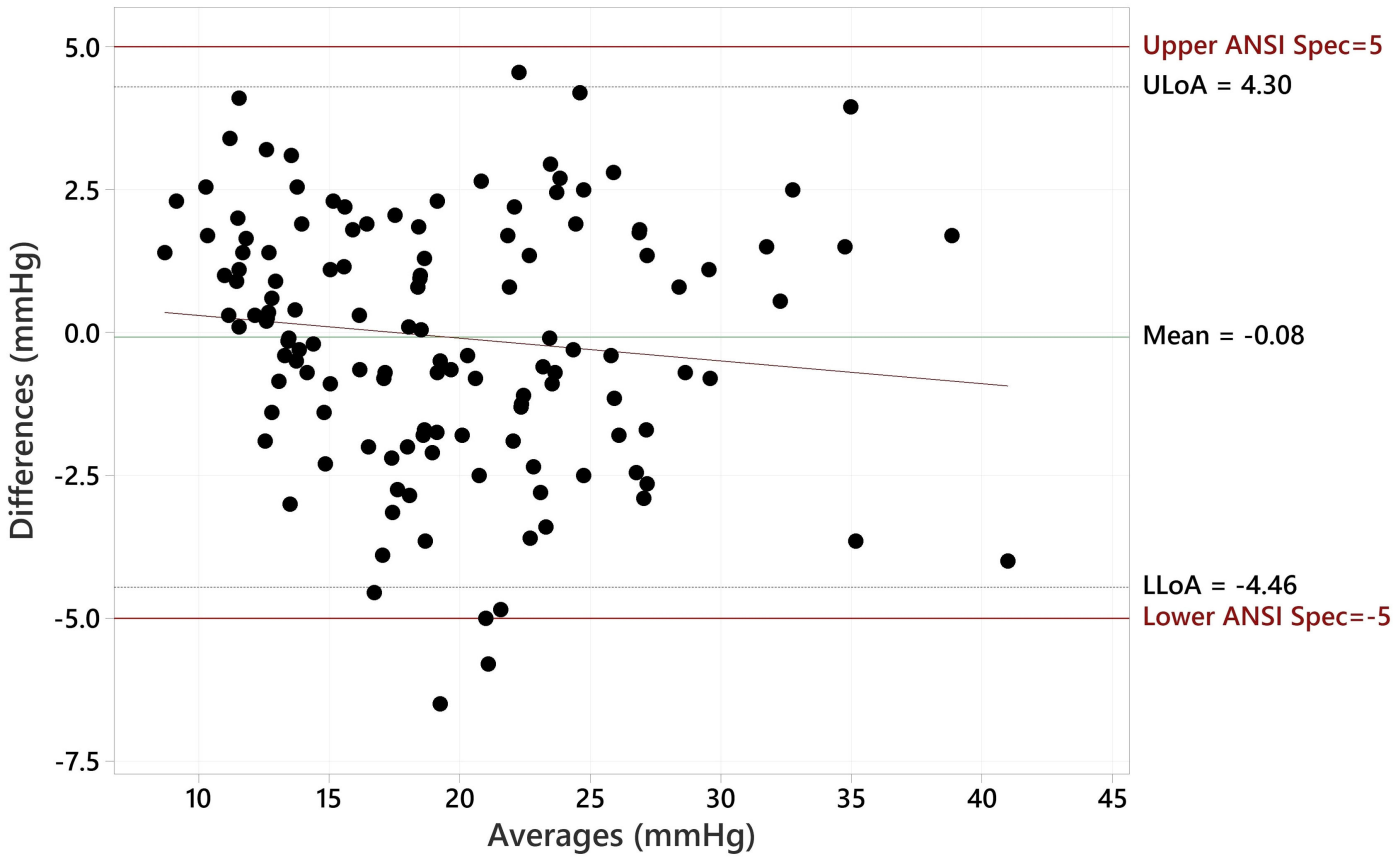
Bland-Altman Comparison of Tono-Vera to GAT for 137 low corneal astigmatism eyes.

**Figure 6:**
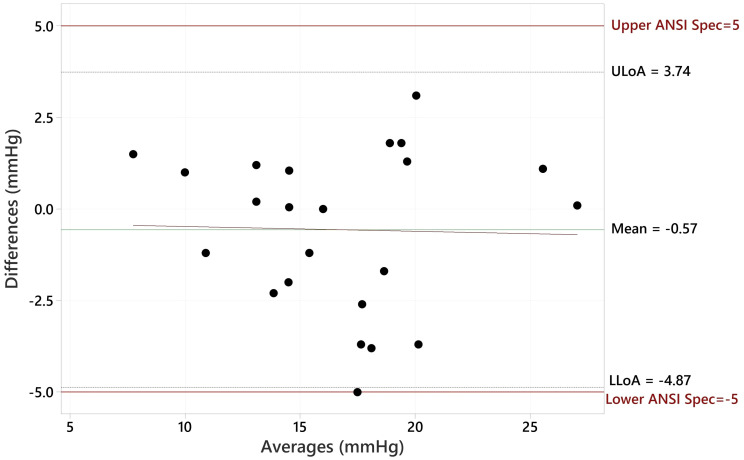
Bland-Altman Comparison of Tono-Vera to GAT for high corneal astigmatism eyes.

The Bland Altman plot for all data, showing the paired differences between GAT and Tono-Vera measurements, shows a mean difference of -0.15 ± 2.23 mmHg (**Figure 4**). **Figure 5** shows the paired differences of the average IOP between the GAT and Tono-Vera for the 137 eyes with low corneal astigmatism. The comparison shows a mean difference of -0.08 ± 2.23 mmHg. **Figure 6** is the Bland-Altman plot for the 23-eye high astigmatism subgroup showing mean difference of -0.57 ± 2.20 mmHg.

Despite substantial efforts to recruit 10 patients with >23 IOP and >3D astigmatism eyes, only two such subjects were identified. Subjects who meet these criteria, and who do not meet any of the exclusion criteria, are extremely rare (23, 24). Nonetheless, our analysis has shown that there is no difference in either tonometer’s performance between the low and high astigmatism eyes in this study. The agreement between GAT and Tono-Vera in eyes with >3D astigmatism compared to eyes with <3D astigmatism was not statistically different when comparing the means of the measurement pair differences between Tono-Vera and GAT (p=0.335) using a 2-sample t-test. As such, we are confident that the results obtained from the available data are sufficient to demonstrate good agreement between GAT and Tono-Vera in eyes with greater than 3D astigmatism.

**Figure 7:**
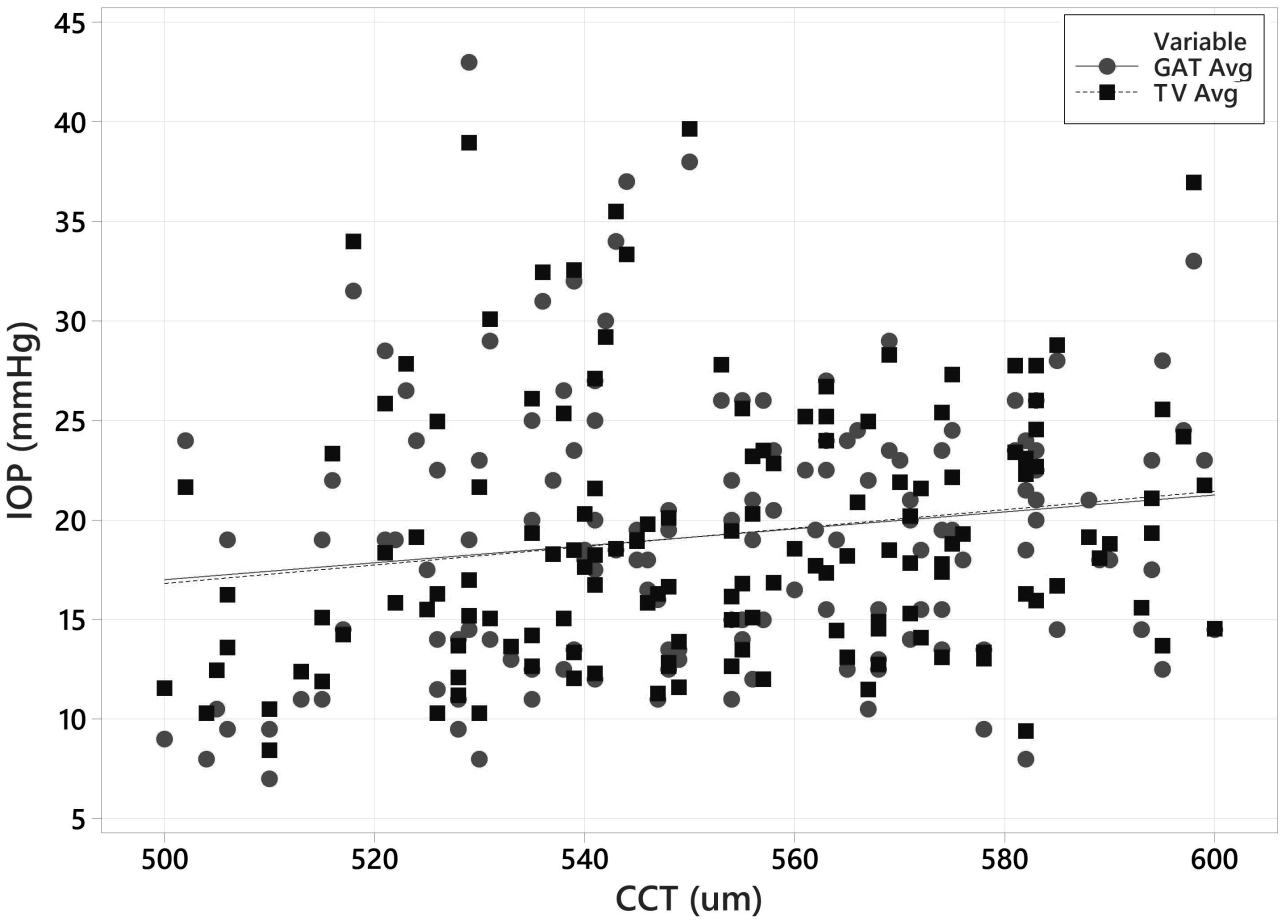
Scatter Plot of GAT & Tono-Vera vs CCT for all 160 eyes.

GAT vs CCT and Tono-Vera vs CCT are plotted together in **Figure 7**. Neither Tono-Vera nor GAT were strongly correlated with CCT in this data set (r^2^ = 0.028 and 0.022 respectively), as would be expected due to the limited range of corneal thickness values required by the ANSI Z80.10 and ISO 8612 standards.

## Discussion

Goldmann Tonometry has been in use for over 6 decades and is found in most eye care practices globally. Despite this, most practices employ multiple tonometry modalities for a wide variety of valid reasons. GAT requires a skilled operator and instillation of topical anesthetic drops. GAT takes more time to execute than other tonometry methods and can be difficult to perform on a significant portion of patients.

Although GAT is considered the reference standard that other tonometers are compared to, it is not without flaws. In addition to the well documented influence of corneal thickness, corneal biomechanics, and tear film on Goldmann-derived IOP values, the GAT measurement process is also subjective in nature (8–10, 14, 15). This brings to light one of the most important, but often overlooked, shortcomings of GAT: its accuracy is dependent on operator skill level and technique (25).

This fact is of increasing relevance in the modern era due to the growing utilization of nurse and technician staff to perform various tests, including tonometry. Staff turnover in the current environment is high, making frequent training of new personnel an ongoing challenge. IOP values taken by staff *must be reliable* as it may not be possible for the physician to verify (double-check) the accuracy of recorded measurements in many modern practices. This makes it especially concerning that clinically relevant errors in measurements made by GAT due to operator skill are possible. Also noteworthy, there are many countries where only ophthalmologists are permitted to use GAT, and so it becomes essential to have reliable alternatives that can be used by staff (with little skill and no interpretation required) and trusted by doctors.

In our data set 32% of Tono-Vera Measurements were within 1 mmHg of GAT, 66% within 2 mmHg of GAT, and 84% within 3 mmHg of GAT. Presumably, the Tono-Vera automated alignment and measurement technology played a role in its agreement with GAT measurements. The PI found Tono-Vera easy and fast to use and would be comfortable delegating IOP measurements with this device to staff.

Patient acceptance of the Tono-Vera measurement was high with most patients stating that they preferred the Tono-Vera over GAT when questioned. The handheld nature of the Tono-Vera device is permits measurements to be made with patients sitting upright, which may reduce transient IOP elevations related to patient posture when in position for GAT measurement. The Tono-Vera measurement probes are single-use, eliminating the need for disinfection procedures. Patents also expressed appreciation of the single-use probes, which may have been top-of-mind considering this trial was conducted during the COVID-19 Pandemic.

Our study had several limitations. We evaluated the agreement of Tono-Vera and Goldmann Applanation Tonometry in eyes with a less than 2-standard deviation range of corneal thickness values. This was necessary in order to comply with the ANSI Z80.10-2014 standard but does not cover the expected range of corneal thickness values in a typical clinical population. In addition, contact lens wearers, post-refractive surgery eyes, and eyes with ocular pathology (other than Glaucoma) were not evaluated in our study. Again, this was necessary to comply with the requirements of ANSI Z80.10-2014 but is not reflective of all eyes in a normal clinical population. Further studies on Tono-Vera vs GAT in normal eyes that have a wider range of corneal thickness values and in eyes with various pathologies or prior surgeries are necessary. We did not directly assess intra or inter operator repeatability of Tono-Vera in our study.

In conclusion, in this study of IOP measurements in a population of patients with a wide range of intraocular pressures, Tono-Vera showed excellent agreement with Goldmann Applanation Tonometry and demonstrated compliance with the requirements of ANSI Z80.10-2014 and ISO 8612-2009. These findings indicate that Tono-Vera can be used to obtain objective and reliable IOP measurements in clinical practice.

## Data Availability

The raw data supporting the conclusions of this article will be made available by the authors, without undue reservation.
