# Supplementary material for: Evaluation of agreement of IOP measurements by Tono-Vera tonometer to Goldmann applanation tonometry
Source: Front Ophthalmol (Lausanne). 2024 Aug 16;4:1441343. doi: 10.3389/fopht.2024.1441343 (PMC11362087; doi:10.3389/fopht.2024.1441343)
Supplement: Supplementary file 1 [file Supplementaryfile1.pdf]

Evaluation of agreement of IOP measurements by Tono-Vera Tonometer to Goldmann Applanation Tonometry.

Supplementary Material

Diagram of device:

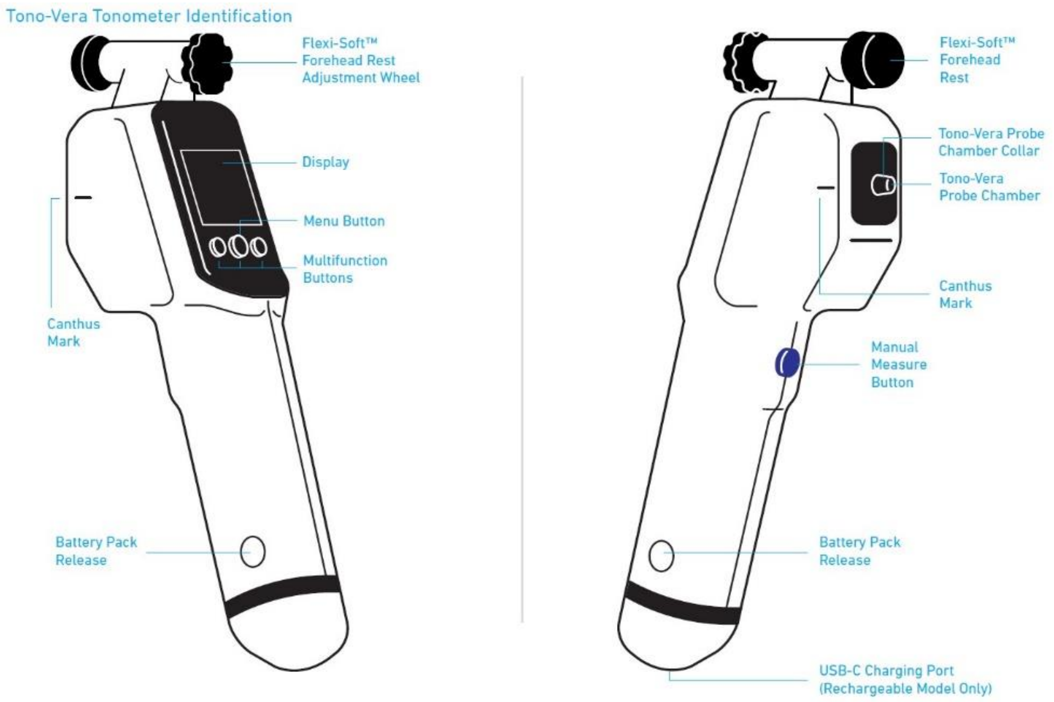

Photo of device:

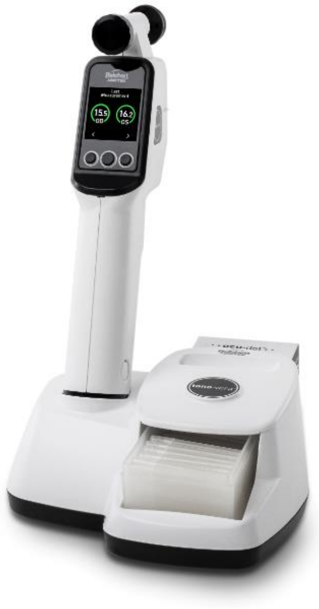

Video of Tono-Vera: https://www.youtube.com/watch?v=jtLT5fZOSw4
